# Oral anticoagulant therapy and outcome in patients with stroke. A retrospective nation‐wide cohort study in Austria 2012–2017

**DOI:** 10.1002/pds.5296

**Published:** 2021-06-03

**Authors:** Jasmina Preinreich, Safoura Sheikh Rezaei, Martina Mittlböck, Stefan Greisenegger, Berthold Reichardt, Michael Wolzt

**Affiliations:** ^1^ Department of Clinical Pharmacology Medical University of Vienna Vienna Austria; ^2^ Center for Medical Statistics, Informatics, and Intelligent Systems Medical University of Vienna Vienna Austria; ^3^ Department of Neurology Medical University of Vienna Vienna Austria; ^4^ Austrian Health Insurance Fund, Burgenland Eisenstadt Burgenland Austria

**Keywords:** drug utilization, NOAC, pharmacoepidemiology, stroke, VKA

## Abstract

**Purpose:**

Patients with stroke are at high risk of recurrence of vascular events. Non‐vitamin K oral anticoagulant (NOAC) and vitamin K antagonists (VKA) are used as secondary prophylaxis. The aim of this study was to evaluate the utilization of NOAC and VKA, and their impact on re‐stroke or death in Austria.

**Methods:**

We analyzed retrospective data between 2012 and 2017 from medical services covered by the health insurance funds, which provides health care for all residents in Austria. Patients without anticoagulant medication 3 months preceding the index event were eligible.

**Results:**

76 354 patients were discharged with a hospital diagnosis of stroke. From these, 16 436 patients with a median age of 78 years received VKA or NOAC. After adjustment, the recurrence of stroke was less frequent in patients with NOAC compared to those with VKA (HR 0.87; 95%CI 0.77–0.97). However, there was no difference in mortality rate after adjustment for age, sex, and co‐morbidities for patients with NOAC (HR 1.0; 95%CI 0.92–1.08). Diabetes (HR 1.25, 95%CI 1.08–1.45; HR 1.25, 95% CI 1.13–1.38) and cardiovascular disease (HR 1.43, 95%CI 1.24–1.65; HR 1.27, 95%CI 1.16–1.39) were significantly associated with re‐stroke or death. Younger age (*p* = 0.0028; HR 0.99, 95%CI 0.99–0.99) was significantly associated with re‐stroke, and advanced age (*p* < 0.0001; HR 1.09, 95%CI 1.08–1.09) with death.

**Conclusion:**

NOAC prescription is related with a reduced risk of re‐stroke but increased mortality compared to patients with VKA. The event risk is associated with diabetes, cardiovascular disease and age.

1

**Key Points**

- In addition to primary prevention, oral anticoagulation therapy in patients with stroke is associated with additional protection against future cerebrovascular events in our real life analysis.
- The risk of recurrent stroke and patients survival depends on the treatment choice, especially between non‐vitamin K oral anticoagulant and vitamin K antagonists.
- Co‐morbidities such as chronic kidney disease, respiratory disease, and cardiovascular disease as well as diabetes are associated with patients outcome.

## 2 INTRODUCTION

Vitamin K antagonists (VKA) and non‐vitamin K oral anticoagulants (NOAC) are used for prevention of stroke and systemic embolism in patients with atrial fibrillation. Over the past few years, the NOAC dabigatran, rivaroxaban, apixaban, and edoxaban have become available for clinical use and offer several advantages over VKA.^1^ However, in some cases careful dosing is required to avoid an increased bleeding risk.^2^

Patients with a history of stroke are at high risk of recurrence of vascular events including stroke. The resulting morbidity, mortality, and health care costs drive the prevention of these events as an important goal in the disease management of these patients.^3^ While controlled clinical trials have demonstrated the efficacy of treatment with VKA or NOAC in primary or secondary prevention, these studies do not necessarily reflect real life and long‐term effectiveness of anticoagulant treatment in the general population after a stroke event.

The aim of this retrospective epidemiological study was to evaluate the utilization of NOAC and VKA, and their impact on re‐stroke or death in anticoagulant‐naive adult patients discharged with an index‐stroke diagnosis in Austria between 2012 and 2017.

## 3 METHODS

### 3.1 Data preparation

Data from medical services covered by health insurance funds are stored in the respective databases, which include demographic data, information on hospital discharge diagnoses and reimbursed drug prescriptions. Each drug is described by the unique Austrian pharmaceutical registration number, which is linked to the Anatomical Therapeutic Chemical (ATC) Classification System. We analyzed data between 2012 and 2017 from Austrian health insurance funds covering about 98% of the Austrian population. Data storage and handling were in agreement with data protection laws. Patient data were pseudonymised to preserve patients' privacy.

### 3.2 Cohort selection

Patients aged 18 years or older with a hospital discharge diagnosis of stroke or TIA (ICD code: G45, I61, I63, and I64) were eligible for this study. ATC codes and sub‐codes B01AF01, B01AF02, and B01AE07 identified patients with NOAC, and B01AA including sub‐codes those receiving VKA, respectively. Only patients with discharge diagnosis of stroke with no anticoagulant therapy 3 months prior to the index event and a medication intake in the first 3 months after the index event were considered for further analysis. Diabetes, arrhythmia, cardiovascular (CV) disease, impaired renal function, respiratory disease, ventricular septal defect, gastrointestinal (GI) bleeding, and thrombosis and embolism were defined as co‐morbidities. ACT codes and sub‐codes for these comorbidities are listed in the Appendix 1.

### 3.3 Statistical methods

The continuous variable age was described by median, minimum and maximum. Categorical data are described by absolute frequencies and percentages. Survival estimates for overall mortality are shown by Kaplan–Meier curves and groups are compared by a log‐rank test. Cumulative incidence curves are shown for the competing events of re‐stroke and death. Cox regression model is used to estimate effects of OAKs, age, sex, and co‐morbidities on re‐stroke or death in an univariate or multiple manner. Covariate effects are quantified by hazard ratios (HR) and corresponding 95% confidence intervals (95% CI). All *p*‐values ≤0.05 were considered statistically significant. Statistical analysis was performed by the statistical software SAS® (Version 9.4, ©SAS Institute Inc., Cary, NC).

## 4 RESULTS

**FIGURE 1:**
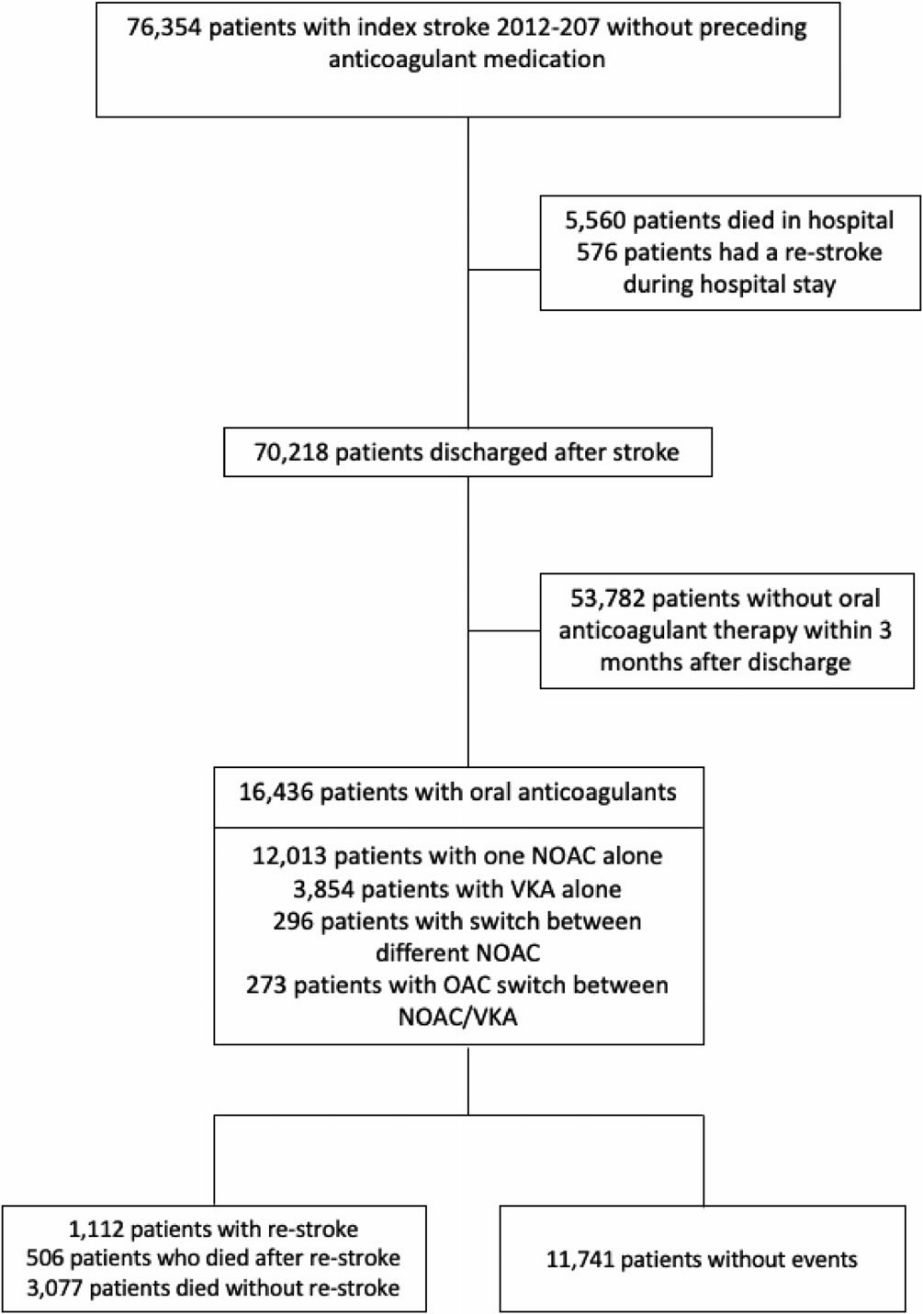
Flow chart

76 354 patients with an index‐stroke diagnosis between 2012 and 2017 were identified. From these, 16 436 patients with a median age of 78 years (range, 18–103 years) were discharged with VKA or NOAC (Figure 1). Table 1 presents patient characteristics and co‐morbidities at index‐stroke diagnosis. The median follow‐up period for patients under study was 30 months with a maximum of 72 months.

**TABLE 1:** Co‐morbidities defined by ICD‐10 codes or concomitant medical therapy in patients with index‐stroke diagnosis who were discharged with VKA or NOAC. Variables are described by median (minimum‐maximum) or number (%)

	Female (*n* = 8662)	Male (*n* = 7774)	
Age (years)	80 (18–103)	75 (20–103)	78 (18–103)
Arrhythmia	2426 (28.0%)	1966 (25.3%)	4392 (26.7%)
Cardiovascular disease	847 (9.8%)	960 (12.4%)	1807 (11.0%)
Diabetes	827 (9.6%)	835 (10.7%)	1662 (10.0%)
Impaired renal function	425 (4.9%)	387 (5.0%)	812 (4.9%)
Respiratory disease	190 (2.2%)	273 (3.5%)	463 (2.8%)
Atrial or ventricular septal defect	81 (0.9%)	106 (1.4%)	187 (1.1%)
Medical history of thrombosis and embolism	81 (0.9%)	49 (0.6%)	130 (0.8%)
Gastrointestinal bleeding at index‐stroke	26 (0.3%)	26 (0.3%)	52 (0.3%)

### 4.1 Anticoagulation treatment after index‐stroke

In total, 4127 patients with VKA (25.1%; 3854 [23.4%] with VKA prescription alone) with a median age of 75 years (range, 18–98, for both groups), 3620 with dabigatran (22.0%; 3332 [20.3%] with dabigatran alone) with a median age of 76 years (range, 22–99, for both groups), 4411 with rivaroxaban (26.8%; 4104 [25.0%] with rivaroxaban alone) with a median age of 79 years (range, 24–103, for both groups), 4672 with apixaban (28.4%; 4424 [26.9%] with apixaban alone) with a median age of 80 years (range, 24–102, for both groups), 190 with edoxaban (1.2%; 153 [0.9%] with edoxaban alone) with a median age of 78 years (range, 30–99 or 46–99, respectively for the two groups) were retrospectively observed for occurrence of an endpoint after index‐stroke. 569 (3.5%) prescription switches between anticoagulant medicines were noted.

### 4.2 Re‐stroke and overall survival analysis after index‐stroke

**FIGURE 2:**
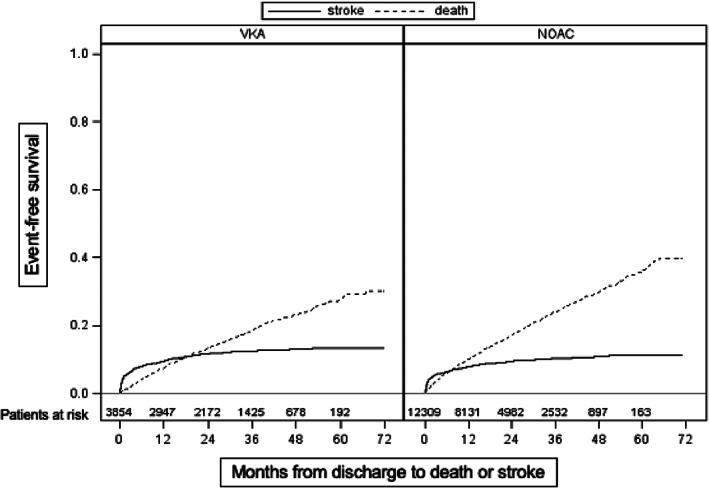
Patient outcome after index‐stroke in patients receiving VKA (left panel) and NOAC (right panel). Cumulative incidences for re‐stroke (solid line) and death (dashed line) events are indicated

3077 patients died after discharge without re‐stroke and 1618 were diagnosed with a re‐stroke. From 1618 patients with stroke, 506 patients died. Table 2 and Figure 2 present patient's survival according to their anticoagulant treatment regime (VKA or NOAC) during the observation period.

**TABLE 2:** Number of re‐stroke or death during the observation period in patients grouped by anticoagulant treatment

Oral anticoagulant^a^ (*total number*)	Re‐stroke *n*(%)	Death *n*(%)
VKA (*n = 4127*)	491 (11.9%)	762 (18.5%)
Dabigatran (*n = 3620*)	390 (10.8%)	517 (14.3%)
Rivaroxaban (*n = 4311*)	442 (10.0%)	1031 (23.4%)
Apixaban (*n = 4672*)	354 (7.6%)	849 (18.2%)
Edoxaban (*n = 190*)	0	11 (5.8%)

^a^ Patients with an oral anticoagulant treatment switch are included in groups of initial OAC treatment.

After adjustment for age, sex, and co‐morbidities the recurrence of stroke remained less frequent in patients with NOAC (HR 0.87; 95%CI 0.77–0.97), followed by the group of patients with oral anticoagulant (OAC) switch (HR 0.88; 95 %CI 0.60–1.29) compared to those with VKA. In contrast, the effect on mortality was no longer statistically detectable after adjustment for age, sex, and co‐morbidities for patients with NOAC (HR 1.0; 95%CI 0.92–1.08) and for those with OAC switch (HR 1.0; 95 %CI 0.77–1.29) compared to patients with VKA (Figure 2).

### 4.3 Co‐morbidities associated with re‐stroke and overall mortality

Diabetes (*n* = 1662 [10.0%]; *p* = 0.0031, HR 1.25, 95%CI 1.08–1.45), CV disease (*n* = 1807 [11%]; *p* < 0.0001; HR 1.43, 95%CI 1.24–1.65), medical history of thrombosis and embolism (*n* = 130 [0.8%]; *p* = 0.0240; HR 1.63, 95%CI 1.07–2.49), and younger age (*p* = 0.0028; HR 0.994, 95%CI 0.989–0.998) were significantly associated with re‐stroke. Impaired renal function was associated with re‐stroke (*n* = 812 [4.9%]; *p* = 0.0777; HR 1.20, 95%CI 0.98–1.47), but not on a statistically significant level. There was no significant association between sex, arrhythmia, respiratory disease, GI‐bleeding, or ventricular septal defect and re‐stroke.

**FIGURE 3:**
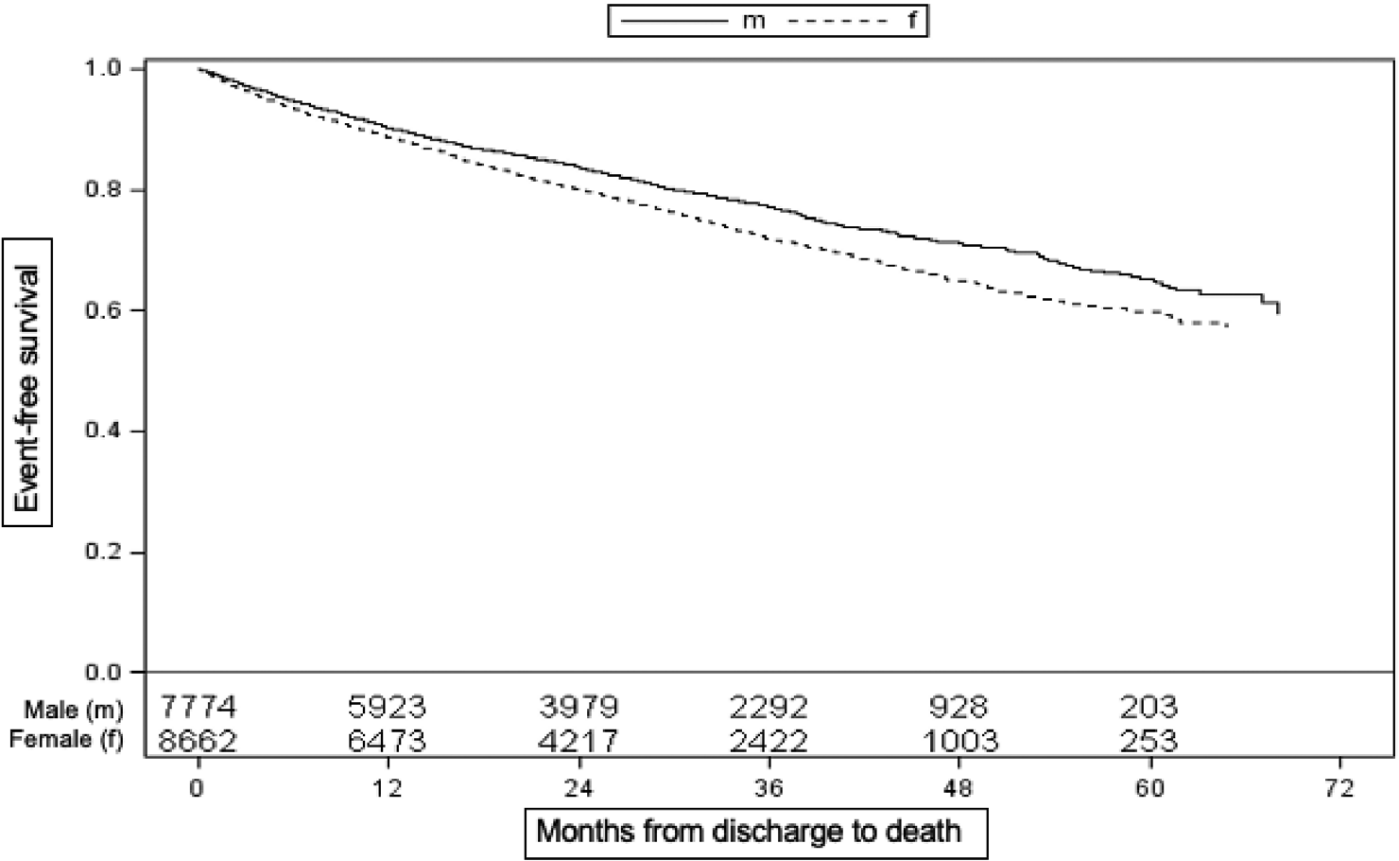
Overall survival of patients grouped by sex (male: solid line, female: dashed line). Event curves indicate re‐stroke or death and are not adjusted for age

Mortality was significantly associated with advanced age (*p* < 0.0001; HR 1.086, 95%CI 1.081–1.09), diabetes (*n* = 1662 [10.0%]; *p* < 0.0001; HR 1.25, 95%CI 1.13–1.38), CV disease (*n* = 1807 [11.0%]; *p* < 0.0001; HR 1.27, 95%CI 1.16–1.39), respiratory disease (*n* = 463 [2.8%]; *p* < 0.0001; HR 1.44, 95%CI 1.23–1.68), and impaired renal function (*n* = 812 [4.9%]; *p* < 0.0001; HR 1.34, 95%CI 1.19–1.50). Female patients (*n* = 8662) had a significant lower overall mortality rate compared to males after adjustment for age (*p* = 0.0013; HR 0.89, 95%CI 0.84–0.96), whereas without adjustment older female patients died earlier compared to younger male patients (Figure 3). Patients with ventricular septal defect had a lower overall mortality (*n* = 187; *p* = 0.0440; HR 0.59, 95%CI 0.36–0.99) compared to other patients. There was no association between arrhythmia, thrombosis and GI‐bleeding and mortality.

### 4.4 Subgroup analysis of oral anticoagulant treatment associated with re‐stroke and overall mortality

Patients with dabigatran (HR 1.15, 95%CI 1.02–1.28) and VKA (HR 1.20, 95%CI 1.08–1.34) had a significantly higher risk for re‐stroke when compared to those without the respective OAC treatment. Patients with apixaban (HR 0.77, 95%CI 0.68–0.86) had a significantly lower risk for re‐stroke compared to those without the respective OAC. Rivaroxaban (HR 0.99; 95%CI 0.88–1.10) had no effect on re‐stroke, when compared to patients without this OAC. In the multivariate analysis, when taking all OAC simultaneously into account, only patients with VKA (HR 1.38, 95%CI 1.09–1.75) and dabigatran (HR 1.34, 95%CI 1.60–1.70) had a significantly increased risk for re‐stroke. Edoxaban was not part of this analysis due to the small number of prescriptions and the lack of re‐stroke events.

Patients with rivaroxaban (HR 1.24; 95%CI 1.15–1.32) and apixaban (HR 1.27, 95%CI 1.18–1.37) had a significantly increased risk of mortality, whereas patients with dabigatran (HR 0.75, 95%CI 0.68–0.81) and VKA (HR 0.80, 95%CI 0.74–0.86) had a significantly decreased risk of mortality, when compared to patients without the respective OAC. Edoxaban had no effect on the risk of mortality (HR 0.86; 95%CI 0.48–1.56). In the multivariate analysis, when taking all OAC into account simultaneously, only patients with VKA (HR 0.81, 95%CI 0.67–0.98) and dabigatran (HR 0.75, 95%CI 0.61–0.91) had a significantly lower risk of mortality.

## 5 DISCUSSION

In this retrospective population‐based study, we assessed the risk of re‐stroke or death in a large nationwide cohort of patients without anticoagulant medication preceding an index stroke. The group of patients with VKA or NOAC treatment was selected because these medicines are available to mitigate the risk of recurrence of ischemic events, but real life experiences are limited. In our population sample, the presence of atrial arrhythmia was not a prerequisite for anticoagulant treatment.

One of our major findings is that patients treated with NOAC had a smaller risk for re‐stroke, but a higher risk for overall mortality than those receiving VKA. A similar reduction in cerebrovascular events has been shown in a previous observational study in atrial fibrillation patients with intracerebral hemorrhage in Denmark.^4^ Results from previous randomized clinical trials with dabigatran,^5^ rivaroxaban,^6^ or apixaban^7^ are consistent with our findings.

In our retrospective study the survival curve crossed the stroke recurrence curve earlier under NOAC than under VKA (Figure 2). Mortality between each individual oral anticoagulant in our study is in line with findings from the RE‐LY (VKA 36% vs. dabigatran 35% [150 mg] and 41% [110 mg]),^8^, ^9^ ROCKET‐AF (VKA 50% vs. rivaroxaban 48%),^10^ and ARISTOTLE (VKA 42.3% vs. apixaban 45.3%).^11^ However, less than 20% of the study population in these prospective trials had a history of stroke. It should be noted that patients with NOAC were on average older than those receiving VKA. A possible explanation for the reduced overall mortality in patients receiving VKA in our study is that those patients are more closely monitored by scheduled visits for VKA dose adjustment. Notably, our data show that OAK is increasingly prescribed to patients aged over 75 years in Austria. These results are in line with a previous investigation on prescribing patters of OAK in the elderly in Austria, and Denmark.^12^, ^13^

Another major finding in the present study is that after adjustment for age, female sex was associated with a lower risk for mortality, which is in agreement with results from a Swedish registry.^14^ Stroke is well known to be a disease of elderly,^15^ but with increasing prevalence of cardiovascular risk factors in young adults a shift of stroke burden towards younger patients has been discussed.^16^ These findings are in agreement with our results.

In our subgroup analysis, we found a significant difference in the risk of re‐stroke for dabigatran and VKA compared to patient with other OAC. In contrast, a registry study from Norway reported no significant difference in occurrence of re‐stroke when comparing NOAC with VKA in patients with atrial fibrillation.^17^ The lack of information on disease severity may be another reason for this contrast. However, intracranial bleeding was shown to be lower for NOAC when compared with VKA,^17^ which is in line with our results. In contrast to our results, a previous Danish study reported a significantly lower risk for intracranial bleedings for patients with dabigatran compared with VKA.^18^ The observation period in our study was longer, but we had no information on other stroke or bleeding risk factors, compared to the Danish and Norwegian study. It should be noted that dabigatran and rivaroxaban were the first (2008), apixaban the second (2011), and edoxaban the third (2015) NOAC available in Austria. Thus, data on edoxaban were limited due to the short observation period. The prescription charges per package for each individual NOAC are identical in Austria.

Our results from subgroup analysis suggest a higher risk of mortality in patients with rivaroxaban and apixaban compared to patients with other OAC. VKA and dabigatran were associated with a lower risk for mortality when compared to patients with other OAC. However, these results were not corrected for co‐variables such as age, gender, and comorbidities. This effect on mortality should be studied more closely in further trials to detect a potential bias.

Our study has also analyzed the association between co‐morbidities and outcome in patients treated with VKA or NOAC. In the present study diabetes was associated with re‐stroke or death. The difference in patient survival between diabetic and non‐diabetic stroke patients has been shown previously in a Swedish population‐based study (HR = 1.66)^19^ and in a Danish cohort study (HR = 1.74).^20^ Furthermore, our investigation showed an association between impaired renal function and re‐stroke or death, which is in line with findings from a previous study.^21^ It remains unclear if the presence of kidney disease simply reflects the fragility of patient's condition.

The multivariate analysis further revealed an association between risk of death and concomitant respiratory disease. Previous epidemiological studies from Sweden, Germany, and Norway reported a higher prevalence of respiratory diseases in stroke patients (7%, 7.1%, and 3.3%, respectively) compared to the general population (0.8–3.6%).^22^, ^23^, ^24^ The interaction between concomitant respiratory disease and stroke has been discussed before,^25^ but the underlying mechanism has not yet been identified.

The association of cardiovascular disease with re‐stroke and mortality is well established^26^ and has also been demonstrated in our cohort. However, our findings regarding the reduced mortality risk of patients with septal defects may be due to the small number of patients in this subgroup, treatment in specialist care, or subject to chance.

GI‐bleeding is one of the typical adverse events in patients with OAC. In our anticoagulant‐naive population the number of GI‐bleeding was small after initiating treatment with NOAC or VKA and only eight events were reported in total. Since these data were collected retrospectively by using hospital discharge ICD‐diagnoses, we did not have access to information on gastroscopy in out‐patients, nor data on blood transfusion or bleeding severity.

### 5.1 Limitations

There are several limitations in this retrospective observational study. To identify our study population ICD‐10 coding was used, but further information on the severity of stroke or the location of cerebral lesions was not available. The claims database of the health insurance funds does not include clinical information such as laboratory parameter, disease severity, and other ambulatory treatments. Furthermore, there was no recommendation for the treatment with NOAC or VKA related to the severity of the clinical condition. The decision for a particular anticoagulant medicine was made by physicians independently and therefore may be subject to some selection or indication bias Moreover, information on medication dosage and patient's medical adherence were not available.

### 5.2 Conclusion

In this large observational study we found a difference in the risk of re‐stroke and death among anticoagulant‐naive stroke patients receiving NOAC compared to VKA. However, concomitant diseases are associated with clinical outcome and reduce the relative risk difference between treatment regimens.

## CONFLICT OF INTEREST

The authors declared no conflicts of interest.

## ETHICS STATEMENT

This study was approved by the Ethics Committee of the Medical University of Vienna (EK‐No. 1508/2018) and was performed in cooperation with the Pharmacoeconomics Advisory Council of the Austrian Sickness Funds and in accordance with the Declaration of Helsinki.

## Supporting information

Appendix 1: Supporting Information

## Data Availability

Derived data supporting the findings of this study are available from Data Clearing House of Medical University of Vienna on request.

## References

[1] Yeh CH, Hogg K and Weitz JI. Overview of the new oral anticoagulants: opportunities and challenges. Arteriosclerosis Thrombosis Vascul Biol 2015; 35: 1056–1065. 2015/03/21. DOI: 10.1161/atvbaha.115.303397. 25792448

[2] Vranckx P, Valgimigli M, Heidbuchel H. The significance of drug–drug and drug—food interactions of Oral anticoagulation. Arrhythmia Electrophysiol Rev. 2018;7:55‐61. 10.15420/aer.2017.50.1. PMC5889806 29636974

[3] Morris JG, Carter EL and Martin SA. Stroke: secondary prevention of ischemic events. J Family Pract 2017; 66: 420–427. 2017/07/13. 28700757

[4] Nielsen PB, Skjøth F, Søgaard M, Kjældgaard JN, Lip GYH, Larsen TB. Non‐vitamin K antagonist Oral anticoagulants versus warfarin in atrial fibrillation patients with Intracerebral hemorrhage. Stroke. 2019;50:939‐946. 10.1161/strokeaha.118.023797. 30869568 PMC6430592

[5] Connolly SJ, Ezekowitz MD, Yusuf S, et al. Dabigatran versus warfarin in patients with atrial fibrillation. N Engl J Med. 2009;361:1139‐1151. 10.1056/NEJMoa0905561. 19717844

[6] Patel MR, Mahaffey KW, Garg J, et al. Rivaroxaban versus warfarin in nonvalvular atrial fibrillation. N Engl J Med. 2011;365:883‐891. 10.1056/NEJMoa1009638. 21830957

[7] Halvorsen S, Atar D, Yang H, et al. Efficacy and safety of apixaban compared with warfarin according to age for stroke prevention in atrial fibrillation: observations from the ARISTOTLE trial. Eur Heart J. 2014;35:1864‐1872. 10.1093/eurheartj/ehu046. 24561548 PMC4104493

[8] Hart RG, Diener HC, Yang S, et al. Intracranial hemorrhage in atrial fibrillation patients during anticoagulation with warfarin or dabigatran: the RE‐LY trial. Stroke. 2012;43:1511‐1517. 10.1161/strokeaha.112.650614. 22492518

[9] Hankey GJ. Intracranial hemorrhage and novel anticoagulants for atrial fibrillation: what have we learned? Curr Cardiol Rep. 2014;16:480. 10.1007/s11886-014-0480-9. 24643903

[10] Hankey GJ, Stevens SR, Piccini JP, et al. Intracranial hemorrhage among patients with atrial fibrillation anticoagulated with warfarin or rivaroxaban: the rivaroxaban once daily, oral, direct factor Xa inhibition compared with vitamin K antagonism for prevention of stroke and embolism trial in atrial fibrillation. Stroke. 2014;45:1304‐1312. 10.1161/strokeaha.113.004506. 24743444

[11] Held C, Hylek EM, Alexander JH, et al. Clinical outcomes and management associated with major bleeding in patients with atrial fibrillation treated with apixaban or warfarin: insights from the ARISTOTLE trial. Eur Heart J. 2015;36:1264‐1272. 10.1093/eurheartj/ehu463. 25499871

[12] Olesen JB, Sørensen R, Hansen ML, et al. Non‐vitamin K antagonist oral anticoagulation agents in anticoagulant naïve atrial fibrillation patients: Danish nationwide descriptive data 2011–2013. Europace. 2015;17:187‐193. 10.1093/europace/euu225. 25236181

[13] Schuh T, Reichardt B, Finsterer J, Stöllberger C. Age‐dependency of prescribing patterns of oral anticoagulant drugs in Austria during 2011–2014. J Thrombosis Thrombolysis. 2016;42:447‐451. 10.1007/s11239-016-1380-1. 27221106

[14] Eriksson M, Glader EL, Norrving B, Terént A, Stegmayr B. Sex differences in stroke care and outcome in the Swedish national quality register for stroke care. Stroke. 2009;40:909‐914. 10.1161/strokeaha.108.517581. 19118246

[15] Feigin VL, Lawes CM, Bennett DA, et al. Worldwide stroke incidence and early case fatality reported in 56 population‐based studies: a systematic review. Lancet Neurol. 2009;8:355‐369. 10.1016/s1474-4422(09)70025-0. 19233729

[16] Feigin VL, Forouzanfar MH, Krishnamurthi R, et al. Global and regional burden of stroke during 1990–2010: findings from the global burden of disease study 2010. Lancet. 2014;383:245‐254. 10.1016/s0140-6736(13)61953-4. 24449944 PMC4181600

[17] Kjerpeseth LJ, Selmer R, Ariansen I, Karlstad Ø, Ellekjær H, Skovlund E. Comparative effectiveness of warfarin, dabigatran, rivaroxaban and apixaban in non‐valvular atrial fibrillation: a nationwide pharmacoepidemiological study. PloS One. 2019;14:e0221500. 10.1371/journal.pone.0221500. 31449560 PMC6709911

[18] Staerk L, Fosbøl EL, Lip GYH, et al. Ischaemic and haemorrhagic stroke associated with non‐vitamin K antagonist oral anticoagulants and warfarin use in patients with atrial fibrillation: a nationwide cohort study. Eur Heart J. 2017;38:907‐915. 10.1093/eurheartj/ehw496. 27742807

[19] Eriksson M, Carlberg B, Eliasson M. The disparity in long‐term survival after a first stroke in patients with and without diabetes persists: the northern Sweden MONICA study. Cerebrovascular Diseases (Basel, Switzerland). 2012;34:153‐160. 10.1159/000339763. 22907276

[20] Rønning OM, Stavem K. Predictors of mortality following acute stroke: a cohort study with 12 years of follow‐up. J Stroke Cerebrovascul Dis. 2012;21:369‐372. 10.1016/j.jstrokecerebrovasdis.2010.09.012. 21075646

[21] Tsagalis G, Akrivos T, Alevizaki M, et al. Long‐term prognosis of acute kidney injury after first acute stroke. Clinical Journal of the American Society of Nephrology: CJASN. 2009;4:616‐622. 10.2215/cjn.04110808. 19211666 PMC2653666

[22] Yin L, Lensmar C, Ingelsson E, Bäck M. Differential association of chronic obstructive pulmonary disease with myocardial infarction and ischemic stroke in a nation‐wide cohort. Int J Cardiol. 2014;173:601‐603. 10.1016/j.ijcard.2014.03.140. 24704409

[23] Haeusler KG, Herm J, Konieczny M, et al. Impact of chronic inflammatory airway disease on stroke severity and long‐term survival after ischemic stroke‐a retrospective analysis. BMC Neurol. 2015;15:164. 10.1186/s12883-015-0414-1. 26349854 PMC4563919

[24] Anecchino C, Rossi E, Fanizza C, et al. Prevalence of chronic obstructive pulmonary disease and pattern of comorbidities in a general population. Int J Chron Obstruct Pulmon Dis. 2007;2:567‐574. 18268930 PMC2699968

[25] Feary JR, Rodrigues LC, Smith CJ, Hubbard RB, Gibson JE. Prevalence of major comorbidities in subjects with COPD and incidence of myocardial infarction and stroke: a comprehensive analysis using data from primary care. Thorax. 2010;65:956‐962. 10.1136/thx.2009.128082. 20871122

[26] Klijn CJ, Paciaroni M, Berge E, et al. Antithrombotic treatment for secondary prevention of stroke and other thromboembolic events in patients with stroke or transient ischemic attack and non‐valvular atrial fibrillation: a European stroke organisation guideline. Eur Stroke J. 2019;4:198‐223. 10.1177/2396987319841187. 31984228 PMC6960695

