# Supplementary material for: Oral anticoagulant therapy and outcome in patients with stroke. A retrospective nation‐wide cohort study in Austria 2012–2017
Source: Pharmacoepidemiol Drug Saf. 2021 Jun 3;30(10):1332–8. doi: 10.1002/pds.5296 (PMC8453954; doi:10.1002/pds.5296)
Supplement: Supplementary file 1 — Appendix 1: Supporting Information [file PDS-30-1332-s001.pdf]

Diabetes
E10 Insulin-dependent diabetes mellitus
E10.0 Diabetic coma
E10.1 Diabetic ketoacidosis
E10.2 Diabetic nephropathy
E10.3 Diabetic retinopathy
E10.4 Diabetic neuropathy
E10.5 Diabetic angiopathy
E10.6 Diabetic arthropathy
E10.7 Diabetes with multiple complications
E10.8 Diabetes with unspecified complications
E10.9 Diabetes without complications
E11 Non-insulin-dependent diabetes mellitus
E11.0 Diabetic coma
E11.1 Diabetic ketoacidosis
E11.2 Diabetic nephropathy
E11.3 Diabetic retinopathy
E11.4 Diabetic neuropathy
E11.5 Diabetic angiopathy
E11.6 Diabetic arthropathy
E11.7 Type 2 diabetes mellitus with multiple complications
E11.8 Type 2 diabetes mellitus with unspecified complications
E11.9 Type 2 diabetes mellitus without complications
E12 Malnutrition-related diabetes mellitus
E12.0 Diabetic coma
E12.1 Diabetic ketoacidosis
E12.2 Diabetic nephropathy
E12.3 Diabetic retinopathy
E12.4 Diabetic neuropathy
E12.5 Diabetic angiopathy
E12.6 Diabetic arthropathy
E12.7 Malnutrition-related diabetes mellitus with multiple complications
E12.8 Malnutrition-related diabetes mellitus with unspecified complications
E12.9 Malnutrition-related diabetes mellitus without complications
E13 Other specified diabetes mellitus
E13.0 Diabetic coma
E13.1 Diabetic ketoacidosis
E13.2 Diabetic nephropathy
E13.3 Diabetic retinopathy
E13.4 Diabetic neuropathy
E13.5 Diabetic angiopathy
E13.6 Diabetic arthropathy
E13.7 Other specified diabetes mellitus with multiple complications
E13.8 Other specified diabetes mellitus with unspecified complications
E13.9 Other specified diabetes mellitus without complications
E14 Unspecified diabetes mellitus
E14.0 Diabetic coma
E14.1 Diabetic ketoacidosis

E14.2 Diabetic nephropathy
E14.3 Diabetic retinopathy
E14.4 Diabetic neuropathy
E14.5 Diabetic angiopathy
E14.6 Diabetic arthropathy
E14.7 Unspecified diabetes mellitus with multiple complications
E13.8 Other specified diabetes mellitus with unspecified complications
E13.9 Other specified diabetes mellitus without complications

Cardiovascular disease
I10 Essential (primary) hypertension
I11 Hypertensive heart disease
I12 Hypertensive renal disease
I20 Angina pectoris
I20.0 Unstable angina
I20.1 Angina pectoris with documented spasm
I20.8 Other forms of angina pectoris
I20.9 Angina pectoris, unspecified
I22 Subsequent myocardial infarction
I24 Other acute ischaemic heart diseases
I24.0 Coronary thrombosis not resulting in myocardial infarction
I24.1 Dressler's syndrome
I25 Chronic ischaemic heart disease
I25.0 Atherosclerotic cardiovascular disease, so described
I25.1 Atherosclerotic heart disease
I25.2 Old myocardial infarction
I25.3 Aneurysm of heart
I25.4 Coronary artery aneurysm
I25.5 Ischaemic cardiomyopathy
I25.6 Silent myocardial ischaemia
I25.8 Other forms of chronic ischaemic heart disease
I25.9 Chronic ischaemic heart disease, unspecified
I40 Acute myocarditis
I41 Myocarditis in diseases classified elsewhere
I42 Cardiomyopathy
I42.0 Dilated cardiomyopathy
I42.1 Obstructive hypertrophy cardiomyopathy
I42.2 Other hypertrophic cardiomyopathy
I42.3 Endomyocardial (eosinophilic) disease
I42.4 Endocardial fibroelastosis
I42.5 Other restrictive cardiomyopathy
I42.6 Alcoholic cardiomyopathy
I42.8 Other cardiomyopathies
I50 Heart failure
I50.0 Congestive heart failure
I50.1 Left ventricular failure
I50.9 Heart failure, unspecified

Respiratory disease

J44 Other chronic obstructive pulmonary disease
J44.1 Chronic obstructive pulmonary disease with acute exacerbation, unspecified
J44.8 Other specified chronic obstructive pulmonary disease
J44.9 Chronic obstructive pulmonary disease, unspecified
J45 Asthma
J45.0 Predominantly allergic asthma
J45.1 Nonallergic asthma
J45.8 Mixed asthma
J45.9 Asthma, unspecified

Arrythmia

I47 Paroxysmal tachycardia
I47.0 Re-entry ventricular arrhythmia
I47.1 Supraventricular tachycardia
I47.2 Ventricular tachycardia
I47.9 Paroxysmal tachycardia, unspecified
I48.0 Paroxysmal atrial fibrillation
I48.1 Persistent atrial fibrillation
I48.2 Chronic atrial fibrillation
I48.3 Typical atrial flutter
I48.4 Atypical atrial flutter
I49 Other cardiac arrhythmias
I49.0 Ventricular fibrillation and flutter
I49.1 Atrial premature depolarization
Atrial premature beats
I49.2 Junctional premature depolarization
I49.3 Ventricular premature depolarization
I49.5 Sick sinus syndrome
I49.8 Other specified cardiac arrhythmias
I49.9 Cardiac arrhythmia, unspecified

Thrombosis and embolism
I26.0 Pulmonary embolism with mention of acute cor pulmonale
I26.9 Pulmonary embolism without mention of acute cor pulmonale
I74.0 Embolism and thrombosis of abdominal aorta
I74.1 Embolism and thrombosis of other and unspecified parts of aorta
I74.2 Embolism and thrombosis of arteries of upper extremities
I74.3 Embolism and thrombosis of arteries of lower extremities
I74.4 Embolism and thrombosis of arteries of extremities, unspecified
I74.5 Embolism and thrombosis of iliac artery
I74.8 Embolism and thrombosis of other arteries
I74.9 Embolism and thrombosis of unspecified artery

Ventricular septal defect
Q21 Congenital malformations of cardiac septa
Q21.0 Ventricular septal defect
Q21.2 Atrioventricular septal defect

Impared renal function

N17 Acute renal failure
N17.0 Acute renal failure with tubular necrosis
N17.1 Acute renal failure with acute cortical necrosis
N17.2 Acute renal failure with medullary necrosis
N17.8 Other acute renal failure
N17.9 Acute renal failure, unspecified
N18 Chronic kidney disease
N18.1 Chronic kidney disease, stage 1
N18.2 Chronic kidney disease, stage 2
N18.3 Chronic kidney disease, stage 3
N18.4 Chronic kidney disease, stage 4
N18.5 Chronic kidney disease, stage 5
N18.9 Chronic kidney disease, unspecified
N19 Unspecified kidney failure
